# WIMANET: The Power of a Network in Wildlife Malaria Research

**DOI:** 10.1111/1749-4877.12983

**Published:** 2025-04-17

**Authors:** Alfonso Marzal, Kasun Bodawatta, Carolina R. F. Chagas, Nayden Chakarov, Mélanie Duc, Tamara Emmenegger, Martina Ferraguti, Luz García‐Longoria, Rafael Gutiérrez‐López, Ricardo J. Lopes, Josué Martínez‐De La Puente, Swen Renner, Diego Santiago‐Alarcón, Ravinder N. M. Sehgal, Daliborka Stanković, Jenny C. Dunn

**Affiliations:** ^1^ Department of Anatomy, Cell Biology and Zoology, Universidad de Extremadura Badajoz Spain; ^2^ Wildlife Research Group National University of San Martin Tarapoto Peru; ^3^ Section for Molecular Ecology and Evolution, Globe Institute University of Copenhagen Copenhagen Denmark; ^4^ State Scientific Research Institute Nature Research Centre Vilnius Lithuania; ^5^ Department of Animal Behaviour Bielefeld University Bielefeld Germany; ^6^ JICE, Joint Institute for Individualisation in a Changing Environment Bielefeld University Bielefeld Germany; ^7^ Museum Luzern, Department of Zoology Lucerne Switzerland; ^8^ CSIC Doñana Biological Station (EBD) Seville Spain; ^9^ CIBER de Epidemiología y Salud Pública (CIBERESP) Madrid Spain; ^10^ CE3C, Center for Ecology, Evolution and Environmental Change & CHANGE, Departamento de Biologia Animal, Faculdade de Ciências Universidade de Lisboa Lisboa Portugal; ^11^ National Center of Microbiology Carlos III Health institute Madrid Majadahonda Spain; ^12^ CIBER de Enfermedades Infecciosas (CIBERINFEC) Madrid Spain; ^13^ MHNC‐UP Natural History and Science Museum of the University of Porto Porto Portugal; ^14^ Natural History Museum Vienna Vienna Austria; ^15^ Department of Integrative Biology University of South Florida Tampa Florida USA; ^16^ Department of Biology San Francisco State University San Francisco California USA; ^17^ Institute for Multidisciplinary Research University of Belgrade Belgrade Serbia; ^18^ School of Life Sciences Keele University Newcastle‐under‐Lyme Staffordshire UK

**Keywords:** capacity building, COST Action, global collaboration, research network, wildlife malaria and related haemosporidian parasites

## Abstract

The Wildlife Malaria Network (WIMANET) is an EU‐COST‐funded global network of researchers and stakeholders interested in wildlife malaria and related haemosporidian parasites. The network has six working groups covering a diverse range of core topics within wildlife malaria research, focusing on genetics and genomics, species identification, vectors, haematology, communities, and communication. Up to now, the network includes 229 members from 45 countries including Europe, America, Africa, and Asia, but this number is continually growing. This review outlines the aims and goals of WIMANET, providing a summary of activities and plans for each of the six working groups for the next years. The network is open to new members, and we provide details on how both new and existing members can get involved in the network and take part in activities. WIMANET provides a global platform for collaborative and innovative research, and we encourage all members of the wildlife malaria community (and beyond) to take advantage of the opportunities the network offers.

## Graphical abstract

The Wildlife Malaria Network (WIMANET) is an EU‐COST funded global network of researchers and stakeholders interested in wildlife malaria and related haemosporidian parasites. In this paper, we review WIMANET's activities to date. We hope this encourages new members to join the network and motivates both new and existing members to participate in its activities.

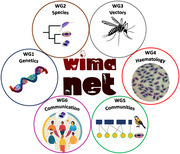

## 1 Background

Malaria and other blood parasites of wildlife are common in nature (Braga et al. 2011; Martinsen et al. 2016; Perkins and Schaer 2016; Bower et al. 2019; Santiago‐Alarcon and Marzal 2020; Fornace et al. 2023). Despite their potential implications for wildlife health and their utility for understanding the evolutionary ecology of vector‐borne diseases, they remain relatively understudied (Rivero and Gandon 2018). Although many research groups worldwide study these parasites, achieving a comprehensive understanding of host–parasite interactions on a global scale requires a multinational and multidisciplinary approach. The Wildlife Malaria Network (WIMANET), funded by the EU Cooperation in Science and Technology (COST), is a research coordination network operating from September 2023 to September 2027. WIMANET aims to unite researchers and research groups studying wildlife malaria and related haemosporidian parasites, to enable a united targeting of specific research objectives across the globe.

WIMANET addresses five specific research coordination objectives, each led by a dedicated working group (WG), alongside a sixth WG responsible for coordinating communication and dissemination activities transversally across all objectives. Research coordination objectives cover a range of key aspects of wildlife malaria ecology and are described in detail below, with each section outlining a specific aim. The objectives range from understanding the genetic insights achievable from whole genome sequencing of intracellular parasites and advancing transcriptomic explorations to establishing databases linking host haematology with wildlife malaria infection status and identifying the vectors of these parasites. Additionally, WIMANET focuses on compiling whole community datasets of host–parasite interactions to identify drivers of host shifts that may contribute to the emergence of these parasites in novel hosts or ecosystems.

WIMANET provides multiple tools to enable the activities of each WG, including funding for travel and capacity‐building activities. Each year, a hybrid workshop/meeting is organised to enable each WG to achieve its goals, the first of which was held at the University of Agricultural Sciences and Veterinary Medicine in Cluj‐Napoca, Romania, from 20 to 23 February 2024 (Gutiérrez‐López et al. 2024a). The second meeting is scheduled from 25 to 28 August 2025 at the Lithuanian Academy of Science in Vilnius, Lithuania, and the third meeting, planned for the spring of 2026, will coincide with the VII International Conference on Malaria and other Blood Parasites of Wildlife, at Universidad de Extremadura, Badajoz, Spain. All future meetings will be advertised through the WIMANET website (https://wimanet‐science.github.io/web/) and on social media. In addition, WIMANET organizes summer training schools to build capacity by transferring expertise among its members. The inaugural summer school took place from 2 to 6 September 2024 at field station Mohelno, Czechia, and the second summer school is scheduled for 18–22 August 2025 at Vilnius University in Vilnius, Lithuania. The first training summer school introduced 22 students to a range of techniques for working with wildlife malaria parasites, including the capture and blood sampling of vertebrates; preparing, fixing and staining blood smears; conducting parasite identification at the genus level by microscopy and calculating host white blood cell differentials from blood smears. Additional activities included the capture and identification of vector species, as well as the analysis of both genetic data and community‐level datasets. The second workshop will focus in more depth on microscopy, focusing on genus‐ and species‐level identification of blood parasites from a range of hosts, alongside the quantification of haematological parameters, and a brief introduction to histological techniques. A third workshop, dedicated to techniques for working with vectors, is planned for the summer of 2026 at Estación Biológica de Doñana, Seville, Spain. Additional online courses will be conducted focusing on the use of bioinformatics for the study of host–parasite–vector interactions, the first of which took place at the end of February 2025.

In addition to organising meetings and training summer schools, WIMANET supports short‐term scientific missions (STSMs), enabling international visits to other research groups for specialised training, specific projects or to establish new collaborations. During its first year, WIMANET awarded eight grants to support STSMs. Furthermore, the network funds attendance at external conferences: through dissemination grants; grants targeted towards younger researchers (aged under 40 years); and grants for researchers in COST Inclusiveness Target Countries (ITCs), which are less research‐intensive COST member countries. More information can be found on the COST website (www.cost.eu), and the WIMANET website (https://wimanet‐science.github.io/web/).

As of December 2024, WIMANET comprises 229 members from 45 countries. Of these, 47% are female, 59% are younger researchers and 50% are based in COST ITCs. In terms of host taxa, the network is rather biased towards researchers working on birds (64% of members) and vectors (across all vector groups; 32% of members). Fewer members specialize in reptiles (6%), mammals (7% comprising: bats [5%], rodents [2%], or primates [0.5%]), amphibians (3%) or fish (0.5%). Many members investigate multiple host groups, while others concentrate exclusively on the parasites or focus on the development of analytical methods. The network is open for new members and is particularly keen to engage researchers specialising in non‐avian host–parasite systems to broaden the WG activities relevant to a wider range of wildlife malaria research areas.

Here, we provide an update on the objectives and current activities of the six WIMANET working groups, as outlined in a keynote presentation at the VI International Conference on Malaria and Other Blood Parasites of Wildlife, held in Medellín, Colombia, from 26–29 November 2024. Additionally, we highlight future research plans, along with opportunities for new and existing members to actively get involved in the network.

## 2 Working Group 1 (WG1): Molecular Markers and Genomics

In recent years, several research groups have been working towards the production of whole genomes for malaria parasites in wildlife (Bensch et al. 2016; Böhme et al. 2018), using multiple methods to overcome the difficulties of delineating the parasite genome from the nuclear genome of its host (Barrow et al. 2019; Videvall 2019). Transcriptomes are also increasingly being used to characterize parasite gene expression (Lauron et al. 2014, 2015; Pauli et al. 2015), identifying some of the molecular mechanisms used by parasites to infect their hosts (Videvall et al. 2017). However, bioinformatics techniques for analysing parasite genomic data remain diverse and potentially incomparable, depending on the pipeline used, and many researchers within the wildlife malaria community currently lack experience with sample handling and bioinformatics approaches.

Therefore, WG1 is currently planning the second in a series of workshops aimed at addressing this knowledge gap. The initial workshop, held online with materials shared after the course, provided an introduction to shell scripting using Unix and employing Galaxy software for workflows (The Galaxy Community 2024), with the aim of introducing the 28 participants to the DNA assembling process. WG1 is also preparing a review article focused on exploring new perspectives on wildlife haemosporidians, opened up by diverse NGS technologies and analytical methods, which will lead to future opportunities.

## 3 Working Group 2 (WG2): Species Identification and Phylogenetic Relationships

WG2 is tackling the challenge of assigning wildlife haemosporidian lineages to species, using both molecular and morphological methods. The scale of this challenge is exemplified by the avian haemosporidians, where although there are over 5000 molecular lineages that have been reported using the cytochrome *b* barcode region (Bensch et al. 2009), fewer than 300 morphological species have been described. The avian haemosporidian field benefits from MalAvi, a curated database collating all avian haemosporidian lineages (Bensch et al. 2009). However, even with careful curation, criteria for morphological identification in parasites with assigned species names, such as some within the *Haemoproteus* genus, are not always clear or convincing, resulting in misidentifications (Shimizu et al., unpublished manuscript). For other genera, such as *Leucocytozoon*, so few genetic lineages have been assigned to morphospecies that any analyses of ecological or phylogenetic associations within morphospecies are challenging due to the paucity of data (Navarrete et al., unpublished manuscript). Data regarding haemosporidian parasites in groups other than birds remain uncurated, and the creation of centralised databases must be seen as the first step towards the development of productive collaborative work. WG2 focuses on improving the rates of morphological description and identification of molecular lineages. The development of an interactive open‐access key, similar in style to the Mos Key Tool (Gunay et al. 2018) and the IIKC *Culicoides* (Mathieu et al. 2012), will enable the development of existing keys (Valkiūnas 2004; Valkiūnas and Iezhova 2022) to include multiple images illustrating the options available for each feature and to prioritize using features that are easily recognised from the parasites visible on any particular slide. The focus of two additional projects within this working group is to enhance knowledge about the key characteristics of haemoparasites that are essential for their morphological identification and the description of new species. The first project involves tutorials aimed at reinforcing the identification and classification of specific features of haemoparasites within host cells. The second project consists of case studies, where participants are provided with images of a haemoparasite to identify from given slides. Both initiatives would greatly benefit from high‐quality images contributed by those involved in microscopy analyses, as well as the appointment of a coordinator for each activity.

## 4 Working Group 3 (WG3): Vector Transmission in Wildlife

WG3 focuses on the vectors of wildlife malaria and related haemosporidian parasites. We are only beginning to understand and identify the key vectors for these parasites (Njabo et al. 2011; Carlson et al. 2015; Chagas et al. 2022; Ferraguti 2024), with different vector groups involved in the transmission of different parasite genera (Valkiūnas 2004). Most studies are currently focused on the identification of parasites in field‐collected dipteran insects (mosquitoes, biting midges, and blackflies), while experimental studies on the vector competence of insect species for the transmission of wildlife malaria and related haemosporidian parasites are very limited. In addition, researchers are starting to identify vector specificity at the species and parasite lineage levels (Martínez‐de la Puente et al. 2011; Gutiérrez‐López et al. 2020, 2024b; Chagas et al. 2022). This emphasizes the complexity of vector–parasite relationships, whose understanding is essential for predicting and quantifying haemosporidian parasite transmission under different environmental scenarios (Ferraguti et al. 2018), especially given the implications of climate change for shifting distributions of haemosporidian parasites in wildlife (Liao et al. 2017; Fecchio et al. 2019).

Working with vectors requires specialised techniques not always familiar to those working with vertebrate hosts—and, for some vector groups, not yet developed. To address this, WG3 is planning a summer school for 2026 focused on vector capture, identification and handling. The WG is also reviewing current protocols for studying the interactions between haemosporidians and other avian blood parasites, hosts and vectors, as well as collating information on laboratory groups with insect colonies and samples to stimulate future collaborative projects. These studies focus on both native and invasive species of vectors, to consider the current global change scenario affecting the transmission of parasites in wildlife, including aspects such as the presence of invasive mosquito species (Veiga et al. 2024).

## 5 Working Group 4 (WG4): Anthropogenic Impacts on Host Haematology

WG4 is examining the interactive impacts of anthropogenic activities and wildlife haemosporidian parasites on their hosts. There are documented cases of malaria parasites having devastating impacts on host populations, such as Hawaiian honeycreepers (Liao et al. 2017; Dahlin and Feng 2019), or penguin species in zoos (Taunde et al. 2019; González‐Olvera et al. 2022). Such events typically occur when parasites or hosts are introduced outside their native range, or when habitats of the hosts are severely altered (Renner et al. 2016; van Hoesel et al. 2020; Ferraguti et al. 2023). Despite medication experiments demonstrating the impacts of malaria and related haemosporidian parasites on host reproduction and survival in co‐evolved hosts (Merino et al. 2000; Marzal et al. 2005; Martínez‐de la Puente et al. 2010), our understanding of the mechanisms underlying these impacts, when they occur, or how malaria parasites interact with other environmental stressors to affect populations, remains limited (Dunn et al. 2013).

WG4 focuses on haematological parameters to assess impacts on hosts. These parameters are widely used as indicators of health and stress in wildlife populations (Davis et al. 2008). Initial work involves compiling a meta‐database of samples (blood smears and blood samples) held by network members to enable future collaborations and analyses. WG4 is also updating existing resources for haematological analyses to encourage capacity building in this research field. Additionally, WG4 enhances with artificial intelligence techniques (Vogelbacher et al. 2024) the automated detection and quantification of white blood cells—and eventually parasites—from wildlife blood smears. These algorithms are currently being trained on avian samples, with the goal of expanding to other host systems following appropriate training on relevant samples.

## 6 Working Group 5 (WG5): Spatiotemporal Variation in Host–Parasite Communities

WG5 aims to scale up the single‐host–single‐parasite relationship paradigm. Increasingly, wildlife malaria studies are now identifying parasite lineages that exist within multiple host species in a community simultaneously, providing valuable insights into both parasite–host generalism and specialism (Ellis et al. 2020; de Angeli Dutra et al. 2021; Woodrow et al. 2023; Bodawatta et al. 2025). The extent to which the environment drives differences in host–parasite associations and host‐switching among different communities is largely unknown (Wolinska and King 2009). However, this understanding may be inferred through the compilation of existing community‐level datasets. Before identifying the factors responsible for these differences, it is necessary to first identify and quantify host–parasite associations across various communities.

The current task of WG5 is to conduct a thorough literature search to generate community‐level host–parasite databases. These databases can then be compiled and analysed to test specific hypotheses pertaining to potential environmental and phylogenetic variation in host–parasite associations. Using community‐level data from the MalAvi database as a benchmark to ensure that suitable papers are not overlooked, literature searches have been conducted to extract studies containing relevant community‐level datasets. The next step is to process these studies and extract and compile relevant datasets for future analyses. Additionally, capitalising on this thorough literature overview, WG5 will identify the methodological and analytical limitations in current host‐parasite community‐level studies. Utilising these insights, we will generate a comprehensive set of guidelines to generate comparable community‐level datasets in future.

## 7 Working Group 6 (WG6): Dissemination and Public Engagement

WG6 focuses on communicating the activities of the network and the achievements of all WGs. This WG has established a WIMANET website (https://wimanet‐science.github.io/web/) alongside a social media presence on Instagram, X (formerly Twitter), YouTube, and Wikipedia. WG6 sends out regular newsletters with updates from WIMANET members, publicising recently published papers and disseminating information about network activities. Additionally, WG6 has produced a brochure and commissioned a professional video introducing WIMANET and its aims (https://www.youtube.com/watch?v=zmbeq6P09CQ). Finally, WG6 has written and published a meeting report from the first in‐person WIMANET meeting, held in February 2024 (Gutiérrez‐López et al. 2024a).

This WG seeks to expand its active membership by recruiting a social media leader to compile and post updates across social media channels, including new accounts on BlueSky (to replace X) and LinkedIn. They also aim to promote the work of WIMANET and its members. Furthermore, they require volunteers to conduct interviews for newsletters and YouTube content, especially focusing on recipients of grants and STSM awards, and to write blog posts and outreach publications.

We hope this review of WIMANET's activities encourages new members to join the network and motivates both new and existing members to participate in its activities. WIMANET serves as a platform to unite researchers and provides financial mechanisms to enable collaboration. We encourage all members of the wildlife malaria community (and beyond) to take advantage of the opportunities the network provides.

## Conflicts of Interest

The authors declare they have no conflicts of interest.
